# Development of Clinical Pathways for Early Diagnosis and Management of SCID, SMA, and XLA Through Newborn Screening in Malaysia

**DOI:** 10.3390/ijns12030045

**Published:** 2026-06-23

**Authors:** Alia Zainudin, Thin Thin Aye, Chloe Chen Sze Yun, Gaayathri Kumarasamy, Adli Ali

**Affiliations:** 1 Department of Pediatrics, Faculty of Medicine, Universiti Kebangsaan Malaysia, Jalan Yaacob Latif, Bandar Tun Razak, Cheras, Kuala Lumpur 56000, Malaysia; aliazndn07@gmail.com; 2 Research Center, Tunku Ampuan Besar Tuanku Aishah Rohani Hospital, Universiti Kebangsaan Malaysia (UKM) Specialist Children’s Hospital, Jalan Yaacob Latif, Bandar Tun Razak, Cheras, Kuala Lumpur 56000, Malaysia; 3 Arcadia Life Sciences, L3-E-10A, Level 3, HIVE 5, Taman Teknologi MRANTI, Bukit Jalil, Kuala Lumpur 57000, Malaysia; thinthin@arcadialifesciences.com (T.T.A.); chloechenszeyun@gmail.com (C.C.S.Y.); gaayathri.tonycheng@gmail.com (G.K.); 4 Institute of IR4.0, Universiti Kebangsaan Malaysia, Bangi 43600, Malaysia; 5 Infection and Immunology Health and Advanced Medicine Cluster, Universiti Kebangsaan Malaysia, Jalan Yaacob Latif, Bandar Tun Razak, Cheras, Kuala Lumpur 56000, Malaysia

**Keywords:** severe combined immunodeficiency, spinal muscular atrophy, X-linked agammaglobulinemia, multiplex PCR assay, newborn screening program

## Abstract

Severe Combined Immunodeficiency (SCID), Spinal Muscular Atrophy (SMA), and X-Linked Agammaglobulinemia (XLA) are rare but life-threatening genetic disorders in infants that can lead to severe infections, progressive neuromuscular degeneration, or severe immune dysfunction associated with significant morbidity and mortality if not diagnosed early. Advances in newborn screening (NBS) technologies have enabled pre-symptomatic detection of these conditions, allowing early initiation of life-saving interventions such as hematopoietic stem cell transplantation, gene therapy, and immunoglobulin replacement therapy. However, the absence of a standardized national clinical pathway linking screening, confirmatory testing, and specialist referral in Malaysia continues to contribute to delayed diagnosis and suboptimal patient outcomes. This review examines and synthesizes current evidence on the clinical pathways for early diagnosis and management of SCID, SMA, and XLA, with particular emphasis on diagnostic workflows, screening technologies, and healthcare system challenges within the Malaysian context. The review examines disease epidemiology, consequences of delayed diagnosis, and the role of expanded NBS under the Screening for Health, Intervention, Nurturing of Every Child (SHINE) program in improving early diagnosis and management. In addition, the paper outlines the current NBS landscape, the use of multiplex real-time polymerase chain reaction (PCR) assays for simultaneous detection of T-cell receptor excision circles (TREC), kappa-deleting recombination excision circles (KREC), and survival motor neuron 1 (SMN1) gene deletion of exon 7 from dried blood spot (DBS) samples. A structured diagnostic framework incorporating screening interpretation, confirmatory testing, and urgency-based referral pathways is also proposed. By addressing current operational barriers and coordinating laboratory referral systems, expanding NBS programs could significantly improve early diagnosis and long-term outcomes for infants affected by SCID, SMA, and XLA in Malaysia.

## 1. Introduction

Severe Combined Immunodeficiency (SCID), Spinal Muscular Atrophy (SMA), and X-linked Agammaglobulinemia (XLA) are rare genetic disorders. Where SCID and XLA compromise immune function by increasing susceptibility to life-threatening infections, SMA leads to progressive motor neuron loss and irreversible muscle weakness in infants and young children. SCID represents the most severe and inherited form of primary immunodeficiency, with a group of genetic defects that impair lymphocytes development [1]. The infants may seem healthy at birth but within the first few months of life, the clinical signs or repeated symptoms like pneumonia and chronic diarrhea with recurrent infections frequently show up [2]. The affected infants often show poor growth or failure to thrive and hypoplastic or absent lymphoid structures, including tonsils and lymph nodes, and may experience severe reactions to routine vaccinations. Symptoms typically grow worse, with increasing suspicion of Pneumocystis carinii pneumonia, cytomegalovirus, or aspergillus infection [3]. Because of this, infants affected by SCID should be treated immediately with prophylactic medication and supportive care to reduce the risk of bacterial, fungal, and viral infections while waiting for treatment [4]. A definitive treatment like hematopoietic stem cell transplantation (HSCT) remains the established curative therapy for most forms of SCID, although gene and enzyme replacement therapy are available for selected genetic SCID subtypes. In Malaysia, the first case of SCID was reported in 1993 [5]. Although SCID remains underdiagnosed, cases continue to be identified through improved clinical recognition and diagnostic testing, with seven cases in 2013 and five cases reported in 2023 [6]. Based on approximately 480,000 annual live births in Malaysia and an estimated incidence of 1 in 58,000 live births [7], approximately eight new cases are expected annually in Malaysia [6]. The first systematic review conducted in Malaysia reported that the prevalence of primary immunodeficiency (PID) in Malaysia was 0.37 per 100,000 population from 1979 until 2019, with 22 patients identified with SCID [8]. Collectively, these findings highlight a significant underdiagnosis of SCID in Malaysia, as evidenced by the clear difference between reported cases and the expected annual incidence.

SMA is an inherited disorder that primarily affects the nervous system. It is caused by the degeneration of alpha motor neurons in the spinal cord, leading to progressive muscle weakness and functional decline. This disorder occurs when individuals inherit pathogenic variants from both parents [9]. There is no curative therapy for SMA. The U.S. Food and Drug Administration (FDA) has approved three disease-modifying therapies, including onasemnogene abeparvovec-xioi, gene replacement therapy (Zolgensma, Novartis); nusinersen, antisense oligonucleotide therapy (Spinraza, Biogen); and risdiplam, the survival motor neuron 2 (SMN2) splicing modifier (Evrysdi, Roche). These gene therapies have significantly altered the natural history of SMA and are an important determinant of survival. Similarly, SMA diagnosis in Malaysia reports an estimated incidence of 1 in 20,000 live births, though comprehensive population-based epidemiological studies remain limited in the region [9]. XLA predominantly affects males in early childhood from two to five years. The estimated global prevalence is approximately 1 in 190,000 live male births [10,11]. Clinical infections and complications such as repeated bacterial pneumonia are most commonly studied in Malaysia [9], followed by chronic lung disease, diarrhea, arthritis, and autoimmune conditions, which are often delayed [5]. Standard XLA care management in Malaysia is typically initiated with intravenous immunoglobulin (IVIG) and subcutaneous immunoglobulin (SCIG) to prevent recurrent infections and long-term complications and to compensate for the immune system’s inability to produce antibodies [12]. Immunoglobulin replacement therapy (IgRT) requires long-term management for XLA and in Malaysia, IVIG is the preferred and widely practiced route [13].

In Malaysia, health professionals often have little familiarity with or understanding of diagnostic tests for patient management [14], or follow-up treatments have resulted in program abandonment or failure to commence [15]. Nordin et al. [16] stated those with other pediatric specialties handling these cases more frequently misdiagnose, due to lack of specialized clinical immunologists in the region. In addition, there is inadequate medical and laboratory personnel to handle the additional workload: East Malaysia’s 1:1700 overall doctor–patient ratio is considerably lower than the WHO-recommended 1:600 [15]. This contributes to parents having difficulty getting accurate knowledge and sometimes dealing with healthcare professionals with inadequate awareness regarding rare diseases [14]. Additionally, Ch’ng et al. [9] reported that caregivers often felt invalidated by healthcare professionals, received insufficient information, and experienced dismissal following an SMA diagnosis. Advances in NBS and molecular diagnostics now offer an unprecedented opportunity for pre-symptomatic diagnosis [9]. The integration of these platforms into a national expanded NBS represents a critical paradigm shift, providing a ‘second chance’ for infants with life-threatening conditions like SCID, SMA, and XLA through timely access to definitive and life-saving interventions. Bridging this gap necessitates not just technology readiness, but also healthcare professional knowledge and coordinated screening procedures to identify affected infants before irreversible issues develop. By providing practical recommendations and highlighting challenges, this review seeks to improve clinician awareness, facilitate early detection, and optimize outcomes for infants affected by SCID, SMA, and XLA, thereby contributing to the development of structured and equitable clinical pathways for early diagnosis and management.

Through NBS, early diagnosis for SCID, SMA, and XLA facilitates the rapid initiation of life-saving treatments such as HSCT, IgRT, and gene therapies, resulting in improved survival outcomes and quality of life. Moreover, it has led to a growing cohort of survivors reaching adulthood, shifting the disease burden from infant mortality toward long-term disease management, including initiates of gene therapies, prophylactic antimicrobial use, and optimized transplant planning [13]. SCID patients diagnosed and treated early with HSCT have a more than 90% survival rate compared to when treatment is delayed [17]. Similarly, five-year survival among SCID patients identified through NBS reaches 92.5%, compared with 79.9% in those diagnosed only after the onset of clinical infections [18]. For SMA, detecting a homozygous deletion of exon 7 in the SMN1 gene at birth allows for initial administration of gene therapy treatments before motor neuron loss occurs. This leads to normal motor skills and age-appropriate milestones, whereas delayed therapy causes irreversibly damaged impairment [17]. Early diagnosis, within weeks of birth, improves lung health in XLA patients, and initiating IgRT helps to decrease severity and reduce mortality rates over time [19]. Importantly, the benefits of early diagnosis and initiation of treatment, with the patients treated before the symptom appears, are now increasingly recognized in these three disorders.

Newborn screening (NBS) is a population-based public health strategy designed to identify serious but treatable conditions before the onset of clinical symptoms, enabling timely intervention and reducing disease-associated morbidity and mortality [15,20]. Economic evaluations have consistently demonstrated that NBS programs are cost-effective when early diagnosis is coupled with timely access to effective treatment and follow-up care [21]. The NBS program in Malaysia began with screening for glucose-6-phosphate dehydrogenase (G6PD) deficiency, established in 1980, and congenital hypothyroidism, in 2003. Both screenings utilize cord blood collected at delivery and remain the routine practice for both screenings in the nationwide NBS program [6,15]. A heel prick is used only as an alternative when cord blood is not obtained at birth [6]. According to Chang et al. [6], most healthcare professionals have not been trained in heel pricking for blood spot collection due to long standing cord blood practices, which may present a challenge to the implementation of an expanded NBS program for SCID, SMA, and XLA.

The screening coverage for G6PD in government hospitals and health clinics is considered universal. Essentially, all babies born must have G6PD screening and closely track with institutional delivery rates in government hospitals. Similarly, congenital hypothyroidism screening in government hospitals achieved approximately 95% coverage using cord blood [22]. However, the remaining gap has been assigned to home births, mothers who deliver in private hospital outside of the government screening network, and logistical issues associated with post-discharge follow-up. Infants born in private hospitals may or may not receive G6PD and congenital hypothyroidism screening, depending on the hospital’s policies, as only around 20% of registered private hospitals with birth and maternity facilities participate in sending samples to the laboratory [15].

In addition, a newborn hearing screening program has been implemented as part of NBS program in Malaysia. Unlike G6PD and congenital hypothyroidism screening, newborn hearing screening does not require blood specimen collection and is performed using bedside physiological screening methods via otoacoustic emissions and automated auditory brainstem response testing. This screening program has expanded substantially over recent years, with 253,822 infants screened in 2023 and national coverage increasing from approximately 60% to 80%, although the Ministry of Health continues to target coverage rates of 98–99% [23,24].

## 2. Materials and Method

This narrative review was conducted through a comprehensive search of electronic databases, including PubMed, Scopus, and Web of Science, from database inception through March 2026. The search strategy employed key terms such as ‘newborn screening,’ ‘severe combined immunodeficiency’, ‘spinal muscular atrophy’, ‘X-linked agammaglobulinemia’, and ‘Malaysia.’ To ensure high-quality evidence, the selection was limited to English-language articles focusing on NBS program, clinical management, referral pathways, confirmatory diagnostics, implementation strategies, and healthcare systems for SCID, SMA, and XLA. Moreover, original research articles, review papers, clinical guidelines, expert recommendations, and policy documents were selected as types of articles. Publications that provide information relevant to the objectives of this review were also included. However, publications not related to SCID, SMA, or XLA, not addressing NBS programs, lacking sufficient methodological detail, not translated to English, as well as duplicate records, conference abstracts, and those outside the scope of the review were all excluded.

In addition, Malaysian-based experts in clinical immunology and clinical genetics, as well as researchers, stakeholders, and patient support group representatives involved in NBS and rare disease diagnosis and patient care, were all invited to participate in a multidisciplinary roundtable discussion conducted as part of the SHINE program. The discussion provided contextual insights into establishing clinical pathways, strengthening clinical awareness, importance of early diagnosis and challenges in implementing NBS for SCID, SMA, and XLA. Perspectives gathered from these exchanges also informed considerations regarding diagnostic and treatment pathways, stakeholder collaboration, healthcare system readiness and the feasibility of expanding NBS initiatives within the Malaysian healthcare setting.

## 3. Expanded Newborn Screening Initiative in Malaysia

### 3.1. The SHINE Newborn Screening Program

Expanded NBS programs, particularly for treatable genetic conditions and metabolic disorders, are not mandated in the public healthcare sector. In response to this gap, the SHINE program was introduced and launched in June 2024 as an expanded newborn screening (ENBS) program initiative in Malaysia and Southeast Asia aimed to facilitate the early diagnosis of more than 65 genetic disorders, including SCID, SMA, and XLA, as well as selected congenital and metabolic disorders in infants. The SHINE program aligns with Malaysia’s National Policy on Rare Diseases and broader global efforts to expand NBS coverage. The implementation of the SHINE program reflects regional and collaborative efforts by involving multiple public and private stakeholders to promote awareness of NBS, assist patient advocacy, and improve equitable access to screening, especially for families from lower socioeconomic backgrounds [25]. However, at present, rather than being a fully nationwide NBS program, the SHINE program conducts targeted or pilot-based initiatives, with high participation in urban and tertiary care centers. This initiative also aims to generate local evidence that supports future expansion of NBS program in Malaysia and Southeast Asia. For the next two years, the SHINE program will target screening 20,000 infants across Southeast Asia. Although cord blood remains the routine specimen used for national screening of G6PD deficiency and congenital hypothyroidism, the SHINE program utilizes dried blood spot (DBS) specimens collected by heel prick to facilitate molecular screening for SCID, SMA, and XLA. At present, DBS collection has not replaced cord blood within the national NBS program. Instead, DBS is collected specifically for ENBS program initiatives and pilot programs targeting these conditions that require molecular analysis. Therefore, both specimen types may be collected concurrently in participating centers, depending on the screening objectives.

Against this background, this program focuses on SCID, SMA, and XLA screening using molecular-based techniques. Additional metabolic and congenital conditions are also included depending on the screening panel. This program is the first national public healthcare initiative that follows a public–private partnership model, in which it utilizes dedicated private laboratories partners across Malaysia to improve accessibility for underserved populations through stakeholder engagement and awareness campaigns. Currently, the SHINE program focuses on screening. Expanded genetic testing is primarily performed on request at two different locations, the Institute for Medical Research (IMR) in the Ministry of Health and the Center for Advanced Analytical Toxicology Services (CAATS) at Universiti Sains Malaysia, with services readily accessible via private health care facilities [15]. Those identified through initial screening for SCID, SMA, or XLA are referred for confirmatory or genetic testing. As the centralized national registry for NBS is not yet established, the SHINE program is still developing efforts to improve data collection and tracking of screened infants, as those data may support policy recommendations.

### 3.2. Screening Interpretation and Referral Pathways

The national surveys from Chang et al. [6] identified lack of awareness and insufficiently trained healthcare professionals as major expert-related barriers, emphasizing the need for workshops, curriculum integration, and continuous professional education to strengthen clinician competency in molecular diagnostics. In parallel, advances in molecular diagnostics in the SHINE program have made it possible to screen for all three conditions simultaneously using a multiplex quantitative polymerase chain reaction (qPCR) assay developed by Revvity (Waltham, Massachusetts, USA) [26]. In this approach, SCID is detected through quantification of TREC [27], SMA is identified by detection of the homozygous deletion of exon 7 in the survival SMN1 gene, and XLA through measurement of KREC as a powerful addition or alternative to conventional screening methods, and the potential exists to detect a broad spectrum of genetic screening conditions from DBS. These advancements are helping to reduce the diagnostic delay, facilitate early treatment, and improve long-term outcomes for affected infants. This approach enables efficient implementation of a single multiplex assay, improving both accessibility and reliability of screening. Internal controls are also incorporated to ensure DNA quality and overall assay performance. However, although the screening assays offer high sensitivity for detecting early signs of SCID, SMA, and XLA, confirmatory testing remains a critical step before clinical decision-making and treatment initiation. Effective NBS under the SHINE program relies not only on lab performance but also on synchronized referral platforms and coordinating clinical follow-up for converting early identification into better health outcomes.

**Figure 1:**
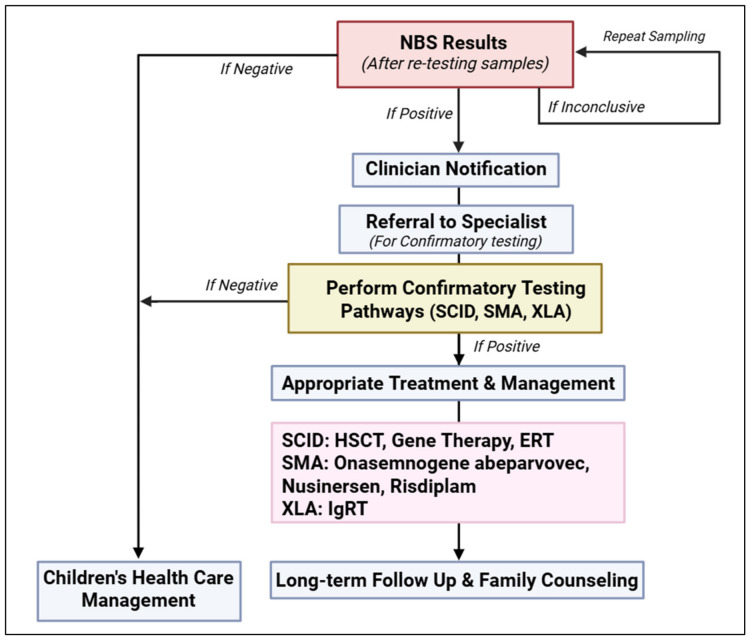
Clinical interpretation, confirmatory pathway, and urgency stratification of SCID, SMA, and XLA.

In the SHINE program, DBS specimens for NBS collected via heel prick are routinely used and processed using multiplex real-time PCR platforms. Pre-analytical factors such as specimen quality, storage conditions, and transportation logistics might affect assay reproducibility and result in false-positive or inconclusive results, just like in all DBS-based assays. Therefore, maintaining diagnostic accuracy requires well-trained laboratory personnel and standardized collection procedures. Results are interpreted using pre-determined cut-off values to determine the presence or absence of the targeted conditions. A diagnostic report is subsequently generated, validated, and submitted to the medical officer for appropriate follow-up actions. Figure 1 illustrates the clinical interpretation workflow and urgency-based referral pathway following multiplex NBS for SCID, SMA, and XLA using DBS samples.

The results of laboratory screening are divided into negative, inconclusive, and presumptive positive. A negative result indicates that the measured TREC, KREC, and SMN1 signals fall within the established reference thresholds. In such cases, no further diagnostic evaluation is required. An inconclusive result is reported when the screening outcome cannot be definitively classified as either negative or presumptive positive. This may occur due to borderline biomarker values, insufficient DNA quality, inadequate specimen integrity, or other technical factors that could affect test interpretation. In these situations, repeat sampling is performed to verify the screening result and minimize unnecessary false-positive referrals. A presumptive positive result requires clinical follow-up and confirmatory diagnostic testing. To ensure result accuracy, an initial presumptive positive finding is subjected to repeat analysis of the original specimen before final classification. Once confirmed by repeat testing, the result is reported as presumptive positive, and the infant is referred immediately for diagnostic evaluation and appropriate clinical management. While this multiplex assay enhances efficiency and scalability, this assay is not designed for screening SCID-like syndromes (e.g., DiGeorge syndrome or Omenn syndrome), nor for detecting less acute SCID variants (e.g., leaky-SCID or variant SCID), not intended for screening B-cell deficiency disorders other than XLA (e.g., atypical XLA), nor for identifying XLA carriers or non-deletion SMN1 variants.

Structured referral pathways to pediatric and immunology specialists for conclusive diagnostic evaluation are initiated by confirmed positive screens. Based on confirmatory findings, conditions such as SCID require immediate referral to specialist immunology centers due to risk of life-threatening infections and for consideration to undergo HSCT, gene therapies or enzyme replacement therapies. Meanwhile, SMA cases require rapid neuromuscular evaluation to initiate disease-modifying therapies as early as possible. Early IgRT and long-term immunology follow-up should be initiated for XLA cases to prevent any recurrent and chronic infections. When clinicians are familiar with the specificity and false-positive rates of newborn screening assays, they can provide accurate counseling to families, reduce parental anxiety, and ensure timely and appropriate confirmatory testing [2]. Long-term follow-up and family counseling should be included for patient management, maintenance of lifestyle changes, and future pregnancy planning for affected families.

### 3.3. Confirmatory Testing Pathways for SCID, SMA, and XLA

**Figure 2:**
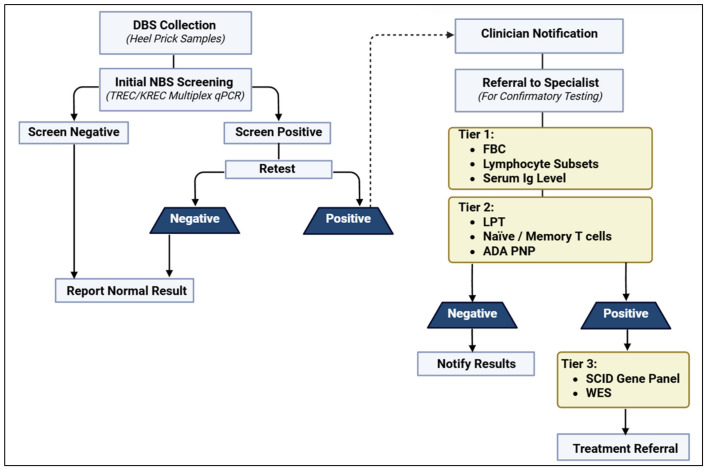
The clinical pathway for SCID and XLA testing, outlining the sequential process from test ordering to confirmatory analysis.

Confirmatory testing is essential to establish a definitive diagnosis before initiating treatment for a positive NBS result. The confirmatory diagnostic process follows a tiered framework comprising initial laboratory assessment (Tier 1), specialized investigations (Tier 2), and genetic confirmation (Tier 3), adapted to the Malaysian healthcare setting. It follows a tiered approach with Tier 1 tests offering rapid and accessible preliminary diagnostics, and Tier 2 tests providing more specific, often molecular-level confirmation and classification of disease subtypes. As shown in Figure 2, initial testing for SCID includes a Full Blood Count (FBC) with a differential to evaluate the absolute lymphocyte count (ALC), as affected infants often present with lymphopenia. Meanwhile for XLA, Tier 1 testing follows a distinct pathway includes flow cytometry to assess B-cell populations, which has revealed a significant reduction or absence of B cells in affected males. In addition, serum immunoglobulin levels like IgG, IgA, and IgM are measured to identify antibody production deficiencies, in spite of the fact that maternally transferred IgG may transiently mask IgG in early infancy, making low IgA and IgM more informative in the neonatal period [7,27].

For SCID, Tier 2 confirmatory testing is performed using a functional T-cell proliferation assay, which assesses the capacity of T cells by measuring their response to mitogens such as phytohemagglutinin. A poor or absent proliferative response is indicative of a profound T-cell defect [28]. In cases where maternal T-cell engraftment is suspected, a phenomenon where maternal T cells cross the placenta and temporarily populate the infant’s circulation then, chimerism testing using short tandem repeat analysis is employed. This method differentiates maternal from infant cells and helps avoid misinterpretation of the T-cell phenotype [29]. It may include the clinical suspicion of metabolic forms of SCID, such as the adenosine deaminase (ADA) enzyme assay measures ADA activity in red blood cells or leukocytes. Meanwhile, the purine nucleoside phosphorylase (PNP) enzyme assay is used to detect PNP deficiency, a much rarer form of SCID associated with T-cell dysfunction and often neurological symptoms. These enzyme assays are useful in guiding both the diagnosis and treatment strategy, including enzyme replacement therapy or ADA-SCID, or consideration of HSCT or gene therapy [27]. In parallel with Tier 2 testing, Tier 3 genetic testing provides treatment planning and precise classification through targeted gene panels or broader approaches such as next-generation sequencing (NGS) increasingly integrated into the workflow after a positive result from Tier 2 testing [30,31]. Identification of mutations in SCID-associated genes and similarly through Bruton tyrosine kinase (BTK) gene sequencing for XLA diagnosis enables targeted carrier testing, family counseling, and donor selection planning. Sanger sequencing or targeted mutation analysis can be performed in female relatives to identify carriers and inform decisions in future pregnancies through prenatal or preimplantation genetic diagnosis. Even though the tiered diagnostic framework provides an organized laboratory strategy and despite its system-specific integration, which includes standardized recommendations pathways, quick reaction times and coordinated access to expert centers is crucial to its efficacy. The entire clinical value of this strategy may be limited in Malaysia due to the variations in the implementation and accessibility.

**Figure 3:**
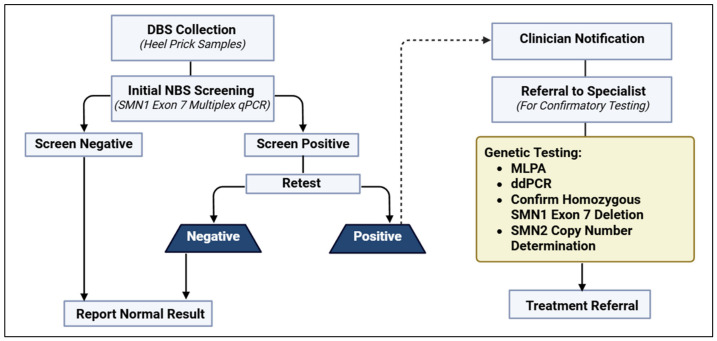
The clinical pathway for SMA testing, outlining the sequential process from test ordering to confirmatory analysis.

Confirmatory testing for SMA shows that approximately 95–98% of SMA patients have a homozygous deletion of exon 7 in the SMN1 gene [32,33]. This analysis can be performed using qPCR, Multiplex Ligation-dependent Probe Amplification (MLPA), or droplet digital PCR (ddPCR). As shown in Figure 3, all of them allow precise quantification and high-throughput detection. MLPA enables precise quantification of SMN1 copy number, identification of the underlying mechanism responsible for SMN1 loss and assessment of SMN2 copy number [26,34]. Several commercial platforms have developed ddPCR-based assays for the quantification of SMN1 and SMN2 copy numbers [35,36]. While these assays provide high sensitivity for typical SMA cases, they do not detect point mutations or compound heterozygous variants. In such cases, whole genome sequencing or targeted NGS panels are required to ensure both diagnostic accuracy and identify pathogenic variants on one allele in combination with a deletion on the other [31,37].

As the molecular diagnostics have expanded the scope and precision of NBS, the value of second-tier NGS integration into NBS has been demonstrated [38]. This approach allows confirmation of specific genetic variants associated with SCID, SMA, and XLA using the DBS-derived DNA, thereby facilitating targeted therapy and management. It also permits the use of less stringent cut-offs, improving the detection while reducing false positives and unnecessary recalls. Importantly, it provides clinically actionable insights that support optimized patient care.

### 3.4. Implementation Challenges and Barriers in Malaysia

As of 2024, Malaysia has had only nine clinical immunologists, with most concentrated in the Klang Valley. The lack of recognition for clinical immunology as a subspecialty further exacerbates challenges in improving care for patient management [13]. The report from Chang et al. [6] also indicates that of the only nine clinicians in Malaysia trained in clinical immunology, one specializes in adult care primarily in the private sector, while the other eight are pediatric immunologists. This contrasts with the recommended ratio of two immunologists per million population. The limited availability of clinical immunologists and specialized centers can contribute to patients undergoing multiple specialist referrals and repeated hospitalizations before receiving an accurate diagnosis [39]. Even in East Malaysia, affected patients are often being misdiagnosed since there are no immunologists. Thus, it is difficult to calculate the exact number of affected populations of SCID, SMA, and XLA due to the absence of a national registry.

Moreover, financial barriers and lack of reimbursement often compel families to cover the high cost of testing and treatment out-of-pocket. NBS coverage in Malaysia remains low, and reported detection rates range from 0% to 35%, which is far below those of high-income countries. Although Malaysia offers a comprehensive range of diagnostic modalities, including biological, genetic, prenatal, and newborn screening, and provides access to genetic and prenatal testing with available treatment (e.g., IVIg, some sub-cutaneous Ig, anti-infectious prophylaxis, and several biological agents), Vietnam remains the only country in Southeast Asia to have implemented a nationwide NBS program for SCID [13]. Meanwhile, for SMA, a major challenge is the inability of current test screening to detect all SMA patients, particularly those with compound heterozygous abnormalities not involving homozygous SMN1 exon 7 deletion, and the high cost of new disease-modifying gene therapies [40]. Identification diagnosis of XLA is also often delayed, with a reported median age of diagnosis of 48 months in Malaysia despite earlier symptom onset [41]. These multifactorial barriers underscore the urgent need for clinician education, coordinated infrastructure strengthening, and equitable resource allocation to optimize NBS early diagnosis of these genetic disorders across the region.

### 3.5. Consequences of Delayed Diagnosis

The journey to establish diagnosis causes significant stress, as a diagnostic delay affects the chances of early HSCT and IgRT. Combined with the absence of immunologists, this leads to frequent hospital visits that are time-consuming and exhausting [39]. Gaviglio et al. [42] also demonstrated that delayed diagnosis significantly worsens outcomes, with a two-year mortality of 71.4% among infants diagnosed after clinical presentation, compared with 29.2% among those identified through NBS. Similarly in SMA, delayed diagnosis results in irreversible motor neuron loss and permanent disability, often due to missed opportunities for timely disease-modifying therapies. Furthermore, FDA disease-modifying therapies for SMA are still not included in Malaysia’s compassionate access program. This reinforces the urgent need for earlier detection and equitable access to multidisciplinary care and approved treatments to improve patient outcomes and quality of life [9]. This reduced eligibility for treatments simultaneously imposes a substantial economic burden on families and healthcare systems due to prolonged hospitalizations and chronic disease management [13]. The financial impact extends beyond direct medical expenses to place sustained strain on healthcare systems. Kumarasamy et al. [1] stated that SCID treatment costs range from USD 50,000 to USD 100,000 in government hospitals and USD 200,000 to USD 400,000 in private hospitals with late detection causing treatment repetition and prolonged hospitalization that further increases costs. In Malaysia’s middle-income healthcare context, this creates a significant coverage gap whereby families may not qualify for full government assistance yet remain unable to afford high-cost specialized treatments, with some parents facing job insecurity, sacrificing work time and social engagements to prioritize caregiving at home [39].

For SMA cases, gene therapy for SMA is highly expensive, representing a significant upfront cost for families and healthcare systems. Introducing combined NBS for SCID and SMA would require an additional USD 35 million from government budgets [17]. The survey study from Ch’ng et al. [9] did not provide a quantitative estimate of the annual cost of care for Malaysian patients with SMA; however, it highlighted the substantial financial burden borne by families, including expenses for medical equipment, medications, physiotherapy, and frequent medical consultations, often necessitating loans and workforce withdrawal of a parent to sustain care. Choo et al. [43] further indicated that, for XLA, tertiary hospital analysis found that 66.7% of IVIG prescriptions were off-label, with 38.5% of total expenditure allocated to lower-evidence indications, thereby contributing to significant cost burdens. Ongoing direct costs remain substantial, as most XLA patients who receive IgRT are subjected to hospitalization or admission into day care units for at least 4–6 h per session [39] and frequent hospital visits contributes to school absenteeism that negatively impacts overall quality of life [1].

The prolonged uncertainty intensifies emotional distress (e.g., anxiety, isolation, and frustration) as symptoms worsen despite repeated medical consultations; therefore, families face increasingly complex treatment decisions and escalating caregiving demands. Meelad et al. [39] added another psychosocial burden is living with fear and anxiety, with caregivers expressing concerns about sickness, burnout, depression, fear of infections, and hereditary risks. In her study, parents and caregivers expressed concern about the 50% chance of passing XLA conditions to male offspring, which affected reproductive decisions. Beyond the physical limitations of SMA, more than 80% of caregivers experience similar mental-health issues, with heightened impact among families of Type 1 patients. Meanwhile, more than half of individuals with SMA (54%) reported experiencing mental health challenges, including stress, anxiety, or depression, with the highest prevalence observed among Type 3 patients (75%), followed by 56% of those with Type 2 SMA [9]. Strengthening clinical pathways for early detection not only improves patient outcomes, but also safeguards the resilience and functional well-being of the family unit.

## 4. Conclusions

The implementation of structured clinical pathways for early diagnosis and management of SCID, SMA, and XLA under the SHINE program represents a pivotal opportunity to improve survival and long-term outcomes for Malaysian children affected by these treatable genetic disorders. The availability of multiple real-time PCR platforms capable of simultaneously detecting from a single DBS provides a practical foundation for nationwide implementation, positioning Malaysia to address the current regional gap [6]. DBS-based screening enables molecular detection of conditions such as SCID, SMA, and XLA, which cannot be reliably identified through the existing cord blood-based screening framework used for routine national screening programs. Adoption of DBS-based screening may promote future extension of national NBS program and align Malaysia with international practices in ENBS program, even though a nationwide switch from cord blood to DBS is not currently underway.

However, NBS must be embedded within a standardized, clinician-driven framework to ensure continuity of care from infancy to adulthood. National referral algorithms linking structured multidisciplinary transition planning beginning in early adolescence, and expansion of adult specialist services capacity are essential [6]. Leong et al. [15] mention that effective NBS implementation relies on multidisciplinary collaboration among pediatricians, obstetricians, neonatologists, nurses and laboratory personnel, policymakers, and patient advocacy groups. The development of standardized protocols and national registries further enhance clinicians’ responsibilities in sample collection, follow-up and confirmatory diagnostic evaluation.

As Malaysia moves from pilot programs to more extensive NBS implementation, addressing workforce limitations and strengthening psychosocial and organizational support systems will be critical to sustainability. Early detection, time management, and equal access to diagnostic and therapeutic treatments can significantly reduce unnecessary morbidity and mortality from SCID, SMA, and XLA, ultimately improving health outcomes for future generations.

## Data Availability

Not applicable.

## Abbreviations

The following abbreviations are used in this manuscript:

ADA	Adenosine Deaminase
ALC	Absolute Lymphocyte Count
BTK	Bruton Tyrosine Kinase
CAATS	The Centre for Advanced Analytical Toxicology Services
DBS	Dried Blood Spot
ddPCR	Droplet Digital PCR
ENBS	Expanded Newborn Screening
FBC	Full Blood Count
FDA	The U.S. Food and Drug Administration
G6PD	Glucose-6-Phosphate Dehydrogenase
HSCT	Hematopoietic Stem Cell Transplantation
IgRT	Immunoglobulin replacement therapy
IMR	Institute For Medical Research
IVIG	Intravenous Immunoglobulin
KREC	Kappa-Deleting Recombination Excision Circles
MLPA	Multiplex Ligation-Dependent Probe Amplification
NBS	Newborn Screening
NGS	Next-Generation Sequencing
PCR	Polymerase Chain Reaction
PID	Primary Immunodeficiency
PNP	Purine Nucleoside Phosphorylase
qPCR	Quantitative Polymerase Chain Reaction
SCID	Severe Combined Immunodeficiency
SCIG	Subcutaneous Immunoglobulin
SHINE	Screening for Health, Intervention, Nurturing of Every Child
SMA	Spinal Muscular Atrophy
SMN1	Survival Motor Neuron 1
SMN2	Survival Motor Neuron 2
TREC	T-Cell Receptor Excision Circles
XLA	X-Linked Agammaglobulinemia
